# Immuno-Imaging (PET/SPECT)–Quo Vadis?

**DOI:** 10.3390/molecules27103354

**Published:** 2022-05-23

**Authors:** Carsten S. Kramer, Antonia Dimitrakopoulou-Strauss

**Affiliations:** 1 Curanosticum Wiesbaden-Frankfurt, Center for Advanced Radiomolecular Precision Oncology, D-65191 Wiesbaden, Germany; 2 Clinical Cooperation Unit Nuclear Medicine, German Cancer Research Center, D-69120 Heidelberg, Germany; a.dimitrakopoulou-strauss@dkfz.de

**Keywords:** immuno-imaging, molecular imaging, immunotherapy, checkpoint inhibitors, drug design, immunoPET, PET/CT, SPECT/CT, response criteria, tumor microenvironment

## Abstract

The use of immunotherapy has revolutionized the treatment regimen of certain cancer types, but response assessment has become a difficult task with conventional methods such as CT/MRT or FDG PET-CT and the classical response criteria such as RECIST or PERCIST which have been developed for chemotherapeutic treatment. Plenty of new tracers have been published to improve the assessment of treatment response and to stratify the patient population. We gathered the information on published tracers (in total, 106 individual SPECT/PET tracers were identified) and performed a descriptor-based analysis; in this way, we classify the tracers with regard to target choice, developability (probability to progress from preclinical stage into the clinic), translatability (probability to be widely applied in the ‘real world’), and (assumed) diagnostic quality. In our analysis, we show that most tracers are targeting PD-L1, PD-1, CTLA-4, and CD8 receptors by using antibodies or their fragments. Another finding is that plenty of tracers possess only minor iterations regarding chelators and nuclides instead of approaching the problem in a new innovative way. Based on the data, we suggest an orthogonal approach by targeting intracellular targets with PET-activatable small molecules that are currently underrepresented.

## 1. Introduction

The use of immunotherapy has revolutionized the treatment regimen of certain cancer types, namely NSCLC [1,2], malignant melanomas [3,4], and renal cell carcinomas [5], but it is also applied in other tumor types such as prostate cancer [6,7], pancreatic tumors [8,9], bladder cancer [10], multiple myeloma [11,12], leukemia [13,14], and lymphomas [15]. The use of CTLA4 as well as PD-L1 and PD-1 inhibitors can be seen as a major milestone in cancer therapy. The therapeutical paradigm shifts also demand a transformation of the diagnostical toolbox. It all comes down to the so vital question ‘is or will the treatment be effective?’—a question as important for the physician who plans therapy as for the physician who assess the treatment success by the tools of medical imaging. Nonetheless, efficacy of new anti-cancer drugs or therapy regimen is often evaluated by clinical endpoints (such as progression-free survival) also applying imaging methods (e. g. CT, MRI or PET/CT) (for a recent review on imaging biomarkers for the evaluation of tumor response, see [16]). Applying RECIST criteria, CT and MRI assessments are routinely used to evaluate the efficacy of new cancer drugs in phase II trials [17], but also FDG-PET is a remarkable tool to clearly discriminate therapy responders from non-responders during chemotherapy [18]. With the rise of immunotherapy, the response assessment for the treating physician or a decision maker in clinical development became difficult based on the classical methods such as CT/MRT or FDG PET-CT and the classical response criteria, such as RECIST or PERCIST which have been developed for chemotherapeutic treatment [19,20,21,22,23,24,25]. The reason is that the response patterns of immunotherapy are different from the known ones of classical chemotherapy. Pseudoprogression, hyperprogression and late response are “new” response patterns observed in patients who receive treatment with immune checkpoint inhibitors. Another problem is caused by immune-related adverse events (irAE). All these aspects cannot be sufficiently answered by the existing response criteria. In particular, the classification of progression is different than in the case of chemotherapy, as one new lesion does not necessarily mean tumor progression after ICI treatment [19].

As the market entry of CTLA4, PD-L1, and PD-1 antibodies, the molecular imaging society made significant efforts to support the evaluation of treatment efficacy and following decision making during therapy with checkpoint inhibitors. The developed imaging tools comprise mainly PET and SPECT tracers, although also MRI contrast agents and NIR probes were published. The work on these imaging agents has been extensively reviewed over the past three years [20,22,26,27,28,29,30,31,32]. Whereby the reviews could summarize the work on key targets (e.g., PD-1, PDL-1, CD8), different imaging modalities (e.g., SPECT, PET, MRI), entities (antibody, peptide, etc.), and different tumor models, we thought it would be necessary to discuss new ways to approach the problem and to evaluate if and how already published work can progress into a product that is reliable and efficient for therapy evaluation. The properties of an immune imaging biomarker for the assessment of immune therapy response can be summarized in four pillars:

1. **Target selection**: a tracer has to address a target found either in *activated immune cells*, that can target cancer cells, or in *cancer cells* that are prone to immune therapy (serving as a tool for pre-treatment stratification). Ideally, the agent should distinguish the desired cytotoxic immune reaction from adverse immune reactions.
2. **Developability**: non-human biologics (such as murine antibodies) might show promising preclinical results in animal studies but could lead to immunogenic reactions in First in Human (FiH) studies. In addition, clearance of tracers in humans can be different from animals.
3. **Translatability**: expensive agents and tracers that require exotic nuclides disqualify themselves from wide application in clinical practice and shift the work-load to specialized centers. Tracers with a slow target enrichment that require more than one patient appointment complicate the patients’ experience due to the necessity of additional late acquisition studies and require the development of dedicated acquisition protocols. Additionally, examinations of those immune cells which would need to be extracted from patients’ blood and re-infused after labeling would be too elaborate.
4. **Diagnostic quality**: the examination needs to support decision making (in clinical practice or clinical development). Needless to say, techniques that offer a higher spatial resolution (such as PET) are likely to be superior. Of course, high sensitivity and high specificity would be the major criteria for diagnostic quality, but to anticipate the outcome, none of the tracers has reached the development stage to allow benchmarking.

## 2. Data Set-Inclusion and Exclusion Criteria

To set the imaging tools developed to date in context with the aforementioned assessment criteria, we have gathered the published tracers and linked them with descriptors that can be used for comparison. The raw data, including all references, can be found in the SI section. Thereby, the following criteria were applied for collection and analysis of the dataset: a tracer is seen as a unique combination of a targeting entity (e.g., mAb, affibody, or peptide), a nuclide, and eventually a chelator. Permutations of chelator (DOTA vs. NOTA) or nuclides (Cu vs. Zr) are seen as different tracers. 

This article has the focus on PET and SPECT tracers suitable and specific for the imaging of immunotherapy. For data acquisition, we screened Scopus^®^ for the following terms: {immunotherapy} AND {PET} AND {tracer}; {immunotherapy} AND {SPECT} AND {tracer}*;* {immunoPET}; {immunoSPECT}; {immunoimaging}; molecular imaging in immuno-oncology. Pubmed was screened using following terms: immunotherapy AND PET AND tracer; immunotherapy AND *S*PECT AND tracer; *i*mmunoPET; immunoSPECT; immunoimaging; molecular imaging in immuno-oncology. Within the identified reviews and original articles, cited literature was screened as well. With broader terms such as immunoPET/immunoSPECT we could also identity tracers that were not intentionally designed for immunotherapy imaging but addressed suitable targets. Relevant articles (in English) were included in our database until 31 March 2021. For clinical studies, we screened clinicaltrials.gov for the following terms: PET AND immunotherapy NOT FDG; SPECT AND immunotherapy. Clinical studies were included in following status: recruiting, enrolling by invitation, suspended, terminated, completed, and unknown status. We included the trial number for the most progressed clinical phase. 

After acquisition, all literature was further refined using the following exclusion and inclusion criteria: articles after deadline as well as articles that describe only the preparation of a tracers were excluded. The focus of our review is on tracers that address targets found either on/in (preferably *activated*) *immune cells* (that for example can target cancer cells) or on/in *cancer cells* that are prone to immune therapy (serving as a tool for pre-treatment stratification, like PD-L1). Due to the aforementioned difficulties assessing treatment response during immunotherapy with FDG, we excluded unspecific ‘conventional tracers’ for metabolization imaging (such as [^18^F]F-FDG, [^18^F]F-FAC, [^18^F]F-CFA, and [^18^F]F-FLT). Targets that were *too ubiquitous* (such as IDO) were excluded from the review, as targeted tracers would be too unspecific (nevertheless, some of these targets might be still useful when linked to other methods (such as FACS or IHC)). Targeting strategies that would require cumbersome isolation of certain immune cell types followed by labeling and re-infusion were also not considered. In addition, markers for chemotherapy-immunotherapy induced apoptosis (such as ([^18^F]-C-SNAT4) were not included in the data set. 

The ‘*immunogenic cascade*’ that is from interest can be seen as: targets that are necessary for the interplay between cancer and immune cells (such as CTLA4, PD-1, and PDL-1), targets that define above mentioned cell types (such as CD8), targets that reflect a successful cytotoxic immune reaction (such as granzyme or IFNγ release, T-cell activation). Nevertheless, the immune system is highly complex and the roles of all involved immune cells are not fully understood. As we wanted to focus on *lymphoid cells* (T cells, natural killer cells), we excluded tracers for myeloid cells (such as tracers of myeloid-specific immune checkpoints (CD47/SIRPα axis); tracers for macrophages (a review on this topic can be found elsewhere [33]); and CD33 as a marker expressed on cells of myeloid lineage. To focus more on targets useful in immunotherapy monitoring, we excluded tracers selective for B cells (such as CD20) and tracers for malignant immune cells such as lymphoma cells. We also excluded imaging methods for reporter genes (e.g., used for CAR-T cell imaging) and imaging methods for transgenic/bioengineered cells. 

Other targets that were not included but are linked to immunotherapy monitoring: EGFR (promiscuous target); P2RY12 (adenosine receptor, promiscuous target); CD11b (promiscuous target); and IDO tracer (promiscuous target). Most publications on CD38 utilize CD38-targeted tracers for imaging of multiple myeloma, but CD38 was recently identified as a possible target for checkpoint inhibition [34], therefore CD38-targeted tracers might also find application in immunotherapy monitoring, but were not included in this review. 

In total, we identified 106 individual SPECT and PET tracers suitable for monitoring subsets of lymphoid cells during immune therapy.

## 3. Analysis

**Target selection**: Our analysis (1a) shows that PD-L1 is the predominant target that is addressed in nearly the half (45%, 48 tracers) of all published tracers, followed by PD-1 (10%), CD8 (9%), and CTLA-4 (7%). All other targets form the residual quarter of the data set. Anti-PD-L1 and anti-PD-1 tracers comprise mainly mAbs (44% for PD-L1, 100% for PD-1) or antibody fragments and protein domains (in the case of PD-L1). Unsurprisingly, as the currently marketed therapeutics against PD-L1 and PD-1 are antibodies, the chance to label those drugs with different nuclides was taken quite often. Despite the dominance of the aforementioned targets, tracers against over 20 different receptors and proteins were published.

**Figure 1:**
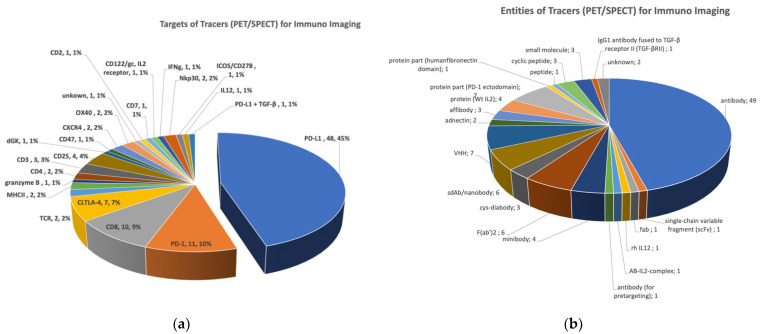
Data analyses of published immuno-imaging reagents (PET/SPECT) (**a**) Representation of targets addressed with selective PET or SPECT tracers. (**b**) Proportional representation the biological and chemical entities.

**Developability**: The strategy to turn to therapeutics in diagnostics goes hand in hand with the fact that 74% of the published tracers are mAbs or their fragments (such as minibodies, f(ab’)2, diabodies, nanobodies, VHHs, etc.) (Figure 1b). To date, only six low molecular weight compounds have been published. The WL12 ligand, a cyclic peptide of 14 AS, binds on PDL-1 and was labelled with gallium-68, copper-64, and fluorine-18 and used in preclinical studies. Two other small molecules were published using ^18^F for PET activation: [^18^F]LN is a PDL-1-inhibitor, whereby [^18^F]F-AraG was found to be a good substrate for dGK and accomplished T cell imaging. N-[^11^C]methyl-AMD3465 is a small molecule PET tracer employing carbon-11 to quantify CXCR4 receptor occupancy. To confirm the use in clinical decision making of these small molecules, clinical translation into humans is needed. In total, low molecular weight compounds (small molecules and cyclic peptides) represent only 6% of the imaging agents. 

**Figure 2:**
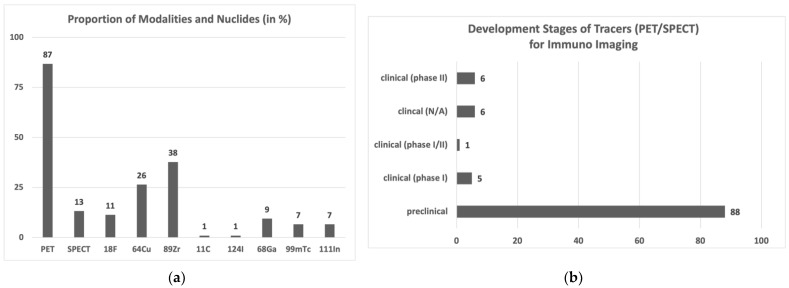
Data analyses of published immuno-imaging reagents (PET/SPECT) (**a**) Proportions of PET vs. SPECT tracers and the applied nuclides in the screened literature. (**b**) Overview of development stages of the published PET/SPECT tracers. Tracers that advanced to clinical stage were not included in ‘preclinical’. Tracers that are tested in different clinical studies (for different tumors, in comparative studies, and in different phases) were counted only once in the most progressed phase.

**Translatability**: As mentioned, nearly three quarters of the published reagents are mAbs or fragments thereof. Those bio-engineered tracers are more expensive than small molecules or peptides. In addition to the bio-engineered scaffolds that are expensive in production, exotic nuclides will further raise the costs as they need to be bought and transported from external production sites if not producible on-site. High costs and limited availability to specialized centers make such combinations (bio-engineered scaffold and exotic nuclide) unattractive for a wide clinical application. Antibodies are also known for undesirable pharmacokinetics, in particular a slow target enrichment and a slow clearance which might require having tracer administration and signal acquisition at different time points to achieve a good tumor to background ratio. This leads to inconvenience for patients and study coordinators. Figure 2a shows that copper-64 and zirconium-89 are the most frequently used nuclides (26% for copper-64, 38% for zirconium-89), whereas the convenient PET nuclide fluorine-18 is underrepresented with 11% (due to the fact of its poor compatibility with slow enriching binders such as mAbs). 

As for some compounds, first-in-human results are already available, and the differences in PK profiles of bio-engineered tracers compared to small molecule tracers can be elucidated. Healthy volunteers could be imaged with the small-molecule tracer [^18^F]F-AraG [35] (targeting dGK) after 47–77 min, while images with the antibody-derived tracers such as [^89^Zr]Zr-nivolumab [36] (PD-1) and [^89^Zr]Zr-*N*-sucDf-atezolizumab [37] (PD-L1) were acquired 7 days post-injection (as the three tracers have different targets, direct comparison of PK profiles has limitations). Concordantly, smaller biomolecules showed faster target enrichments compared to antibodies: SPECT/CT images using the ^99m^Tc-labelled single-domain antibody against PD-L1 [^99m^Tc]Tc-NM-01 could be acquired after 2 h p.i. [38] and with the Tc-labelled recombinant IL-2 (approx. 15 kDa) [^99m^Tc]Tc-HYNIC-IL-2 after 1 h [39]. With the anti-CD8 minibody [^89^Zr]Zr-Df-IAB22M2C, lesion uptake was seen as early as 2 h after injection, but the highest uptake in most lesions was seen at 24 or 48 h p.i. [40]. The tracer [^18^F]F-BMS-986192 could be imaged after 1 h p.i., but this information could not be correlated with the tracer size, as no data about the molecular weight of the PD-L1 targeting adnectin are available [36]. 

Besides the aforementioned technical aspects of translatability, the clinical translation of an imaging biomarker is also important. This means an imaging biomarker should represent underlying biology, for example immune response or the presence of a certain immune cell population. In the preclinical stage, the selection of adequate animal models is important to enable the translation of preclinical findings. The animal models that were used were mostly NSG mice and Balb/c nude mice as immunocompromised/-deficient mouse models, but immunocompetent C57BL/6 mice were also used frequently (a complete list of animal models and their immune status can be found in the SI). The consequences of the use of immunocompromised/-deficient animal models will be discussed later. 

**Diagnostic quality**: To date, several agents against PD-1, PDL-1, CD8, dGK, CTLA-4, CD25 (IL2 receptor), and granzyme B are in clinical phases I or II (see SI), but nearly 90% of the published tracers remain at preclinical stage (Figure 2b). In total, 86% of all published articles are PET tracer, and 83% of all agents tested in human are PET active (not surprisingly, given PET’s superior resolution compared to SPECT). All these tracers might have the potential to monitor therapy response. The question if they impact decision making in clinical practice or clinical studies cannot be finally answered now, but is discussed in the next section.

## 4. Discussion and Further Directions

Figure 2b demonstrates the surplus of tracers in the preclinical stage, a non-surprising fact in drug development. As the most advanced tracers have progressed to phase II, the comparison with the gold standard is still missing. The question to ask is if the ‘holy grail’, which possess all the aforementioned qualities and is able to outperform the current standard (currently, this standard is FDG), can be found within the already published tracers. Starting at the first pillar of our model, the target, it cannot be answered if the best available target has already been found. More comparative clinical data are necessary to provide an answer. One might also ask if the ‘one target approach’ is oversimplifying the biology of the immune system: the detection of the expression of a single biomarker will never give the full picture as the immune system possesses many layers of complexity. However, if other targets shall be evaluated, we suggest switching to tracers based on small molecules, as the use of antibodies and their bio-engineered fragments are limiting the addressable targets to extracellular receptors and often show undesirable properties (immunogenic reactions, slow target enrichment, slow clearance, etc.). Inspiration for small molecules that bind to (intracellular) immune-oncological targets, that were not yet addressed with the published tracers, can be found in the literature [41,42,43]. Luckily, many of these are fluorinated. The continuously ongoing development of new radiofluorination strategies can hereby assist the transformation of therapeutic molecules into new tools for immunoimaging.

Repurposing published small molecules as tracers allows us to use compounds that turned out to be toxic or ineffective in (pre)clinical studies. Saying this, we must stress the importance to publish even negative findings in drug discovery. In addition, small molecules have the potential to be cheaper due to their lower production costs and are often easier to handle due to their stability towards pH and heat. With the tools of medicinal chemistry, small molecules could be structurally modified to undergo preferably renal or hepatic clearance. For example, suppression of hepatic clearance might improve the detection of abdominal lesions (a similar effect was observed when a mAb were exchanged with a f(ab’)2 fragment that possesses lower liver uptake (17)). 

Besides the opportunities of repurposing existing molecules, one major flaw can be seen when turning towards new targets: it is very time- and cost-intensive to generate a lead molecule with a favorable PD and PK profile from scratch—the efforts to develop a small molecule tracer without a lead structure would overstrain an academic lab. However, as many pharmaceutical companies have initiated small molecule programs in immune-oncology, early partnership of academia and industry can lead to attractive collaborations. Academic labs and CROs with specialization in radiopharmacy/nuclear medicine can offer valuable PD/PK data with PET activated lead candidates, and the lead structure might then be further developed into a companion diagnostic that can potentially extend the patent protection [44]. 

While PD-1 and PDL-1 seem to be attractive and feasible targets for small molecule inhibitors, a small molecule binder for CD8—the third most used target in PET imaging—might be challenging to develop as no low molecular weight binders are published. A reason for this could be the lack of therapeutical purpose, as CD8-agonists could lead autoimmune reactions and inhibitors could mitigate the cytotoxic activity against infected cells and tumor cells (besides their potential use against autoreactive T-cells [45]). Elaborate development (HTS, hit to lead, lead optimization) of a small molecule would overwhelm an academic lab and may not give many advantages to the published minibody. 

As the data show, most published tracers remain in the preclinical stage. As no imaging biomarker has progressed to be the gold standard for immune-imaging, one could ask why only a minority of tracers crossed the translational gap and why no tracer has made it into clinical routine to date. One major requirement of any clinically applied imaging biomarker is the reflection of the underlying biology (such as immune response) and that the detected signal can be correlated with the clinical outcome (a roadmap for the development of imaging biomarkers can be found in [46]). Here, an explanation for the limited progress of preclinical tracers might be the inadequate animal models (mostly immunocompromised/-deficient mice) that are not representing the complexity of the human immune system. To ensure that a tracer reflects the biology in the human body well, it becomes necessary to correlate imaging outcomes with histological results from biopsies and consequently with clinical outcomes. In this way, imaging outcome (performed in humans) of the PET tracer [^89^Zr]Zr-Df-IAB22M2C [40] as well as the Tc-labeled anti–PD-L1-sdAb SPECT tracer [^99m^Tc]Tc-NM-01 [38] were correlated with the immunohistochemistry results from surgically removed specimens. Moreover, the uptakes of three different PET tracers were correlated with IHC results of primary tumor samples and, more importantly, with clinical outcomes (therapy response). The uptakes of the PD-L1-targeted tracer [^18^F]F-BMS-986192 and the PD-1-targeted tracer [^89^Zr]Zr-nivolumab correlated well with target expression detected by IHC, and SUV for both tracers were also higher in responding lesions [36]. Additionally, autoradiography and IHC of post-imaging tumor biopsies correlated with the outcome of PET imaging with the PD-L1-targeting tracer [^89^Zr]Zr-*N*-sucDf-atezolizumab, and tracer uptake appeared to be a good predictor for treatment response with atezolizumab (including progression-free survival and overall survival) [37]. As [^18^F]F-AraG [35] was only tested in healthy volunteers, no relationship of tumor uptake in PET with IHC derived from biopsies could be obtained. At the moment, this tracer was intended to detect activated T-cell dynamics during graft vs. host disease, but the compound might be suitable to monitor the effects of immunotherapy. [^99m^Tc]Tc-HYNIC-IL-2 is another candidate for the imaging of activated T lymphocytes during immunotherapy, but to date only its application for atherosclerotic plaques imaging has been reported [39]. 

In conclusion, only clinical studies with a small and often heterogeneous patient collective were published, allowing no direct clinical comparison of the tracers’ performance and utility. At this moment, it is not possible to declare the best tracer or the tracer with the highest potential for immunotherapy imaging. Therefore, stronger-powered and comparative clinical studies are needed to allow final conclusions about the clinical utility and validity of these tracers, and multiple clinical trials are currently ongoing (see Supplementary Materials).

## 5. Conclusions

In summary, despite the huge efforts in the development of immune-imaging agents, no tracer has reached the status of a ‘gold standard’ for monitoring of immunotherapies. We believe that research strategies should also in other directions. Efforts needs to be shifted towards the development of tracers on the basis of small molecules. In combination with easily accessible fluorine-18, which also has superior imaging properties, an imaging tool could be offered that is easily accessible even for small centers. Even though some agents progressed to clinical stage, more data are needed to evaluate if they can outperform the FDG-PET and/or CT/MRT alone and lead to better clinical decision making. If not, other targets might need to be validated. Again, small molecules offer the opportunity to also address intracellular targets, extending the scope of possible targets. As the next generation of immuno-therapeutics will shift from antibodies to small molecules, we see a huge potential for imaging as the new lead structures can be transformed to tracers based on small molecules.

## Data Availability

Data can be found in the Supplementary Materials.

## Supplementary Materials

The following supporting information can be downloaded at: https://www.mdpi.com/article/10.3390/molecules27103354/s1, Excel spread sheet ‘Immuno-Imaging (PET/SPECT)_SI’ with all details of the tracers that were included in this analysis.

## Abbreviations

AB	antibody
[^18^F]F-AraG	2′-deoxy-2′-[18F]fluoro-9-β-D-arabinofuranosylguanine
CD	cluster of differentiation
([^18^F]F-)CFA	2-chloro-2′-deoxy-2′-[^18^F]fluoro-9-β-d-arabinofuranosyl-adenine
CRO	contract research organization
CT	computed tomography
CTLA-4	cytotoxic T lymphocyte associated antigen 4
dGK	deoxyguanosine kinase
DOTA	2,2′,2′,2‴-(1,4,7,10-tetraazacyclododecane-1,4,7,10-tetrayl)tetraacetic acid
Df	desferrioxamine
DFO	desferrioxamine
([^18^F]F-)FAC	1-(2′-deoxy-2′-[*^18^F*]fluoro-β-D-arabinofuranosyl)-cytosine
([^18^F]F-)FLT	*3*′-*deoxy*-*3*’-[^18^F]*fluorothymidine*
FACS	fluorescence-activated cell sorting
([^18^F]F-)FDG	fluorodeoxyglucose
FiH	first in human
HTS	high-throughput screening
IDO	indoleamine-2,3-dioxygenase
IHC	immunohistochemistry
IL	interleukin
mAb	monoclonal antibody
MRI	*magnetic resonance imaging*
NOTA	1,4,7-triazacyclononane-triacetic acid
NSCLC	non-small cell lung cancer
PD	pharmacodynamic
PD-1	programmed cell death protein 1
PD-L1	programmed cell death ligand 1
PERCIST	positron emission tomography (PET) response criteria in solid tumors
PK	pharmacokinetic
RECIST	response evaluation criteria in solid tumors
SPECT	single photon emission computed tomography
